# Association of Isocitrate Dehydrogenase (IDH) Status With Edema to Tumor Ratio and Its Correlation With Immune Infiltration in Glioblastoma

**DOI:** 10.3389/fimmu.2021.627650

**Published:** 2021-03-25

**Authors:** Daniel Dubinski, Sae-Yeon Won, Maximilian Rauch, Bedjan Behmanesh, Lionel D. C. Ngassam, Peter Baumgarten, Christian Senft, Patrick N. Harter, Joshua D. Bernstock, Thomas M. Freiman, Volker Seifert, Florian Gessler

**Affiliations:** ^1^Department of Neurosurgery, Goethe University Hospital, Frankfurt, Germany; ^2^Institute of Neuroradiology, Goethe University, Frankfurt, Germany; ^3^Neurological Institute (Edinger Institute), Goethe University, Frankfurt, Germany; ^4^Department of Neurosurgery, Brigham and Women’s Hospital, Harvard Medical School, Boston, MA, United States

**Keywords:** immune infiltration, glioma microenvironment, dexamethasone, peritumoral edema, peritumoral edema zone

## Abstract

**Purpose:**

The extent of preoperative peritumoral edema in glioblastoma (GBM) has been negatively correlated with patient outcome. As several ongoing studies are investigating T-cell based immunotherapy in GBM, we conducted this study to assess whether peritumoral edema with potentially increased intracranial pressure, disrupted tissue homeostasis and reduced local blood flow has influence on immune infiltration and affects survival.

**Methods:**

A volumetric analysis of preoperative imaging (gadolinium enhanced T1 weighted MRI sequences for tumor size and T2 weighted sequences for extent of edema (including the infiltrative zone, gliosis etc.) was conducted in 144 patients using the Brainlab® software. Immunohistochemical staining was analyzed for lymphocytic- (CD 3+) and myelocytic (CD15+) tumor infiltration. A retrospective analysis of patient-, surgical-, and molecular characteristics was performed using medical records.

**Results:**

The edema to tumor ratio was neither associated with progression-free nor overall survival (p=0.90, p=0.74). However, GBM patients displaying IDH-1 wildtype had significantly higher edema to tumor ratio than patients displaying an IDH-1 mutation (p=0.01). Immunohistopathological analysis did not show significant differences in lymphocytic or myelocytic tumor infiltration (p=0.78, p=0.74) between these groups.

**Conclusion:**

In our cohort, edema to tumor ratio had no significant correlation with immune infiltration and outcome. However, patients with an IDH-1wildtype GBM had a significantly higher edema to tumor ratio compared to their IDH-1 mutated peer group. Further studies are necessary to elucidate the underlying mechanisms.

## Introduction

Glioblastoma (GBM) patients frequently present with peritumoral edema as diagnosed with preoperative imaging, such as T2 or FLAIR MRI scan. Peritumoral edema in turn often causes severe neurological impairment and remains a challenging factor throughout treatment (1). The peritumoral edema in GBM is considered to be vasogenic and caused by increased vascular permeability as hypoxia induced capillary formations lack functional tight junctions, contain fenestration and irregular basal membrane endothelia (2). The disrupted blood brain barrier (BBB) leads to the extravasation of plasma into the brain parenchyma surrounding the lesion.

Furthermore, the hypoxic core of the GBM, often containing necrosis, results in VEGF secretion which in turn is a crucial mediator of peritumoral edema and disrupted tissue hemostasis. Although several studies have identified the synthetic corticosteroid dexamethasone (DEX) as unbeneficial in terms of survival for edema treatment in GBM, it is still routinely used in clinics for peritumoral edema treatment in GBM (3, 4).

Peritumoral edema has been described as a strong propagator of malignant cell infiltration and several studies have confirmed the negative prognostic impact of high edema to tumor ratios in GBM, although the mechanisms remain unclear (5, 6). Perfusion-weighted imaging has been used to demonstrate 50% reduced regional cerebral blood- volume and flow in peritumoral edema compared to the contralateral white matter (7). We therefore postulated that increased edema (including the infiltrative zone, gliosis etc.) to tumor ratio would influence the lymphocytic and myelocytic tumor infiltration in patients with GBM and could therefore be associated with a poor prognosis.

## Material and Methods

### Patients and Data Collection

For this retrospective analysis an ethical approval was obtained from the ethics committee of the University Hospital Frankfurt, Germany, (Identification number: 20-676). As a non-interventional single-center study no patient consent was necessary.

### Cohort

In total, 162 GBM patients that were treated at the authors’ institution between September 2008 and January 2013 were retrospectively analyzed. The inclusion criteria were tumor resection (stereotactic biopsies were excluded) with the histological confirmation of WHO IV GBM without previous treatment such as radio- chemotherapy for low grade astrocytoma. Further inclusion criteria were the availability of a preoperative cranial MRI with gadolinium enhanced T1 sequences and T2 sequences.

Patient medical charts were analyzed by two neurosurgeons (D.D. and S-Y.W.) and blinded to the preoperative radiological data. Tumor and edema volume were analyzed by an experienced neuroradiologist (M.R.) and two neurosurgeons (P.B. and B.B.) who were blinded to the medical chart data. Patient characteristics that were extracted from the medical chart including the preoperative Karnofsky performance scale (KPS), date of surgery, date of death or date of last contact, date of tumor progression that was defined as the date of cranial MRI with progressive disease according to the RANO (8) criteria and/or the determination of the local interdisciplinary neurooncological tumor board.

### Magnetic Resonance Imaging

Preoperative MRI scans were performed in the department of neuroradiology, Goethe University Hospital Frankfurt at a 3 Tesla Siemens Verio scanner. Gd‐DO3A‐butrol (Gadovist^®^, Bayer Vital GmbH) was administered intravenously (0.2 ml/kg, 0.5-1 ml/sec) and imaging started 9 seconds after administration of the contrast agent.

### Image Analysis

**Figure 1:**
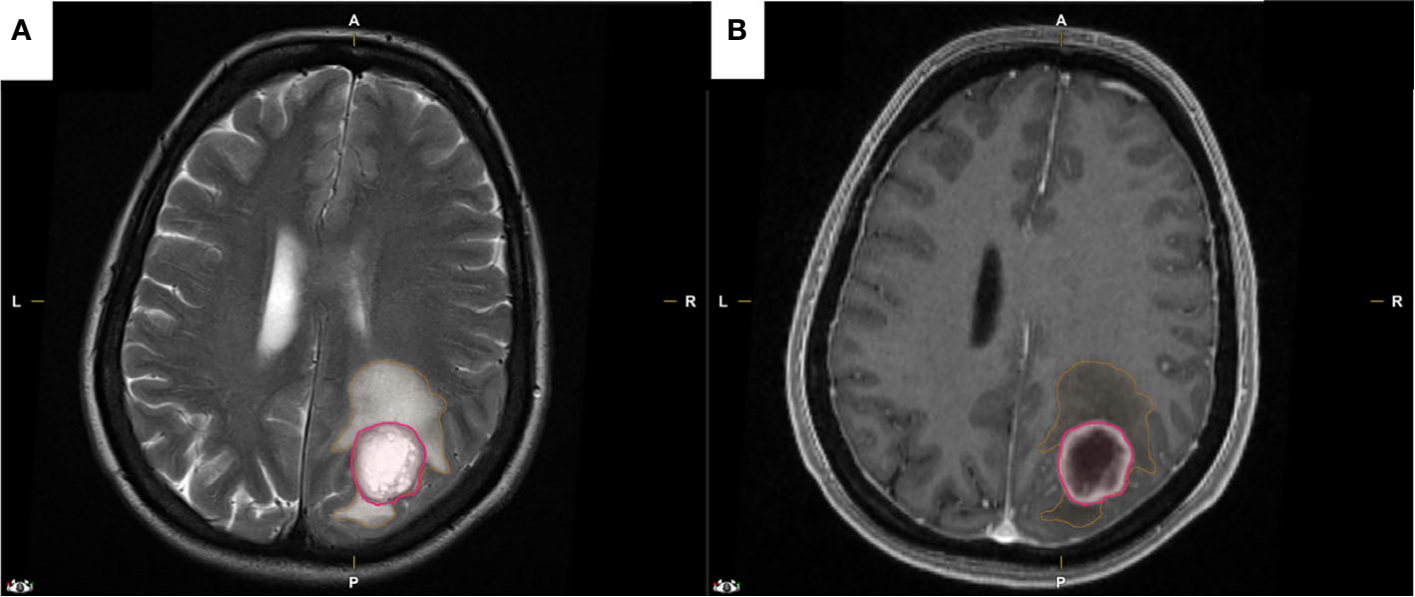
Representative MRI images that were used for the semi-automatic segmentation with IPlannet 3 software by Brainlab®. **(A, B)** Preoperative axial T2 and gadolinium enhanced T1 weighted MRI of a GBM patient with peritumoral edema encircled in orange and with tumor enhancement encircled in pink.

Image analysis was performed by a neuroradiologist (M.R.) and two neurosurgeons (P.B. and B.B.) that were blinded to patients’ molecular characteristics. Pre- and postoperative tumor and edema volumes were analyzed by semi-automatic segmentation with IPlannet 3 (Cranial planning software, Brainlab AG, Feldkirchen, Germany). A representative analysis is displayed in **Figure 1**. All tumor segmentations were done semi-automatically with the ‘Smartbrush’ tool of the Brainlab Elements software. A two-dimensional segmentation was drawn in the axial image and a second two-dimensional segmentation was drawn in a coronal slide. These two segmentations automatically generated a three-dimensional graphic of the tumor. The three-dimensional graphic was then manually corrected by adding or erasing certain areas. A total of 144 segmentations were performed in this manner. Tumor volume was delineated on contrast enhanced T1-weighted images and necrotic areas were spared. Edema volume was measured on a non-enhancing T2-hyperintense image set in the same procedure. The extent of resection was calculated by the pre- and postoperative tumor volume. Gross total resection (GTR) was defined as complete removal (100%) of contrast-enhancing tissue.

### Tissue Specimen and Processing

**Figure 2:**
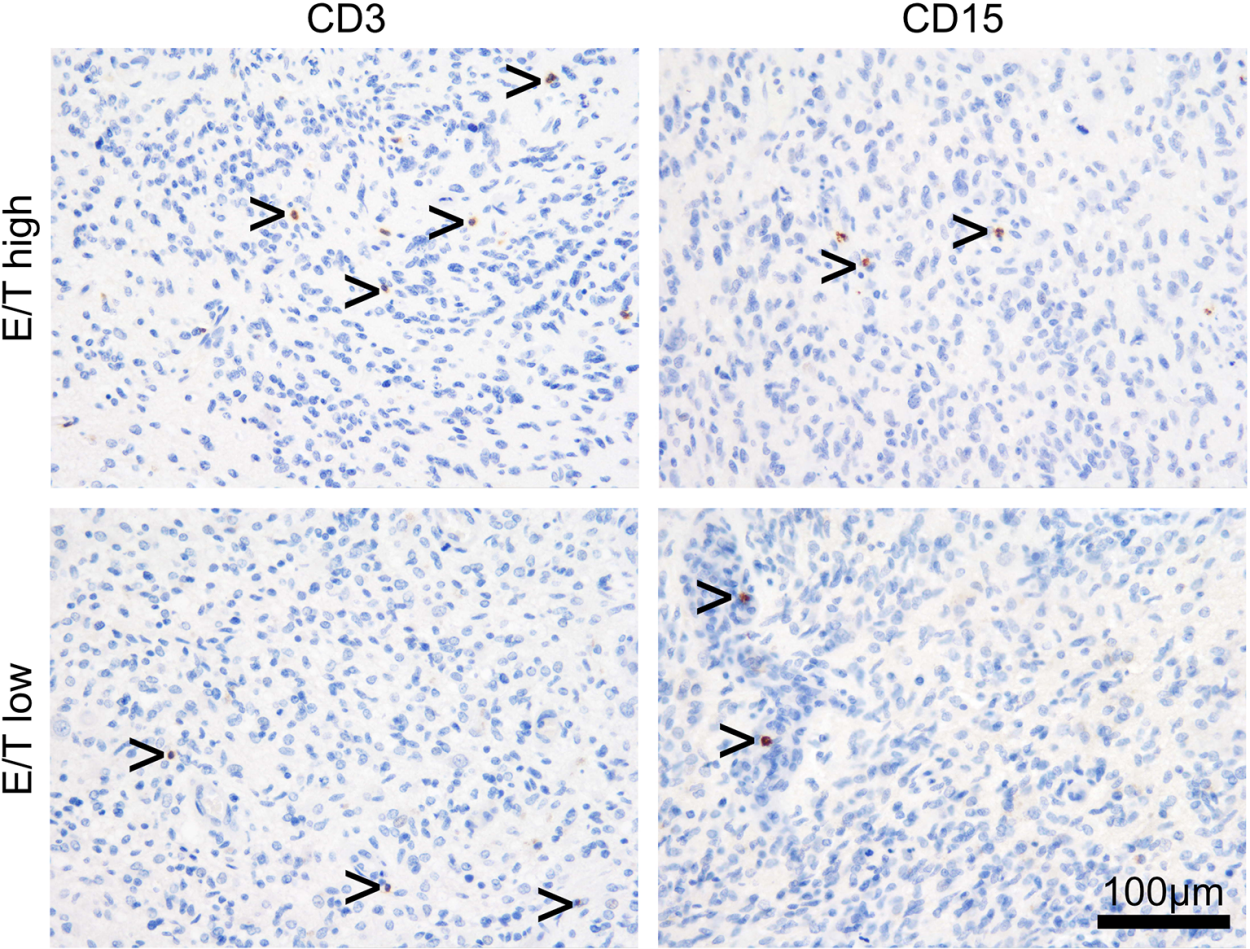
Representative immunohistochemistry slides with anti-CD3 (lymphocytic) and anti-CD15 (myelocytic) tumor infiltration in GBM patients allocated according to high vs low edema to tumor ratio.

For this analysis the formalin-fixed and paraffin-embedded (FFPE) tissue samples from 26 patients of a recently published cohort were used (4). Paraffin full mounts were processed as follows: 1. cutting into 3µm thick slices using a microtome (Leica Microsystems, Nussloch GmbH, Nussloch, Germany), 2. placing on microscope slides (SuperFrost, Thermo Scientific, Dreieich, Germany), 3. heating to 40°C for 20min and 4. storage in an incubator overnight (37°C). For staining, standardized protocols for the automated IHC slide staining system BOND-III (Leica Biosystems, Nussloch GmbH, Nussloch, Germany) were used including the following antibodies: CD3 (A0452; 1:500; Dako), CD15 (M3631; 1:2000; Dako). After hematoxylin-counterstaining slides were mounted, lymphocytic (myelocytic) infiltration was assessed by counting CD3 (CD15) positive stained cells, in a defined tumor bearing area, using a Zeiss microscope (Axiophot, Carl Zeiss Microscopy GmbH, Jena, Germany) with a Stereo Investigator (Version 4.34 software from MicroBrightField Inc.), subsequently obtaining the ratio of positive stained cells per mm^2^ (9). A representative analysis is displayed in **Figure 2**.

### Statistics

Data analysis was performed with IBM SPSS Statistics Version 23.0 (SPSS Inc., IBM Corp., Armonk, NY, USA). For patients and tumor characteristics, descriptive statistics were used. Fisher’s exact test was used for the comparison of categorical variables between the cohorts. For continuous parameters, the Wilcoxon-Mann-Whitney test was used. To assess the impact of the variables, odds ratio (OR) with 95% confidence intervals (CI) were calculated. Results with p ≤0.05 were considered statistically relevant. To estimate the survival rates, the Kaplan-Meier analysis was used. The differences between curves were assessed using the log-rank test. Progression free survival (PFS) was defined as the time from diagnosis to first recurrence or death. Overall survival (OS) was defined as the time of first presentation to death.

## Results

### Patient Characteristics

A total of 144 patients with primary GBM were treated at the authors’ institution between September 2008 and January 2013. Of those, 18 patients were excluded due to a lack of radiological data, loss to follow-up, and/or lack of molecular data. Of the analyzed cohort, 64 patients (44%) were female and 80 patients (56%) were male. Of all patients, 64 patients (44%) were under 60 years of age. In total, 63 patients had a Karnofsky performance scale (KPS) of less than 80 (44%). Preoperative dexamethasone treatment was observed in 57 patients (39%). Gross total resection (GTR) was achieved in 89 patients (62%). O (6)-methylguanine-DNA methyltransferase (MGMT) promotor methylation was detected in 68 patients (47%) and an isocitrate dehydrogenase (IDH) (IDH1R132H-mutation) mutation was observed in 19 patients (13%). The median tumor volume on T1 + contrast preoperative MRI was 29.75 cm^3^ (IQR: 36.6) and median edema volume on T2 was 114 cm^3^ (IQR: 120.8). Median progression free survival (PFS) was 9 months (IQR: 11.5) and overall survival was 17 months (IQR: 19.5). Of the 144 patients with complete diagnostic histopathological analysis, 26 FFPE’s were available for further immunohistochemical analysis. Of those, median tumor infiltration (CD3+) were 0.18/mm^2^ (IQR: 0.15) for lymphocytic (CD3+) cells and 0.08/mm^2^ (IQR: 0.66) for myelocytic (CD15+) cells (see **Table 1**).

**Table 1:** Patient characteristics.

n=144	Number(%)
Gender	
Male	80 (56)
Female	64 (44)
Age (years)	
<60	64 (44)
≥60	80 (56)
Karnofsky performance scale	
<80	63 (44)
≥80	81(56)
Dexamethasone preoperative	
yes	57 (39)
no	87 (61)
Surgical Charactersitic	
Gross totaI resection	89 (62)
PartiaI resection	55 (38)
Tumor Charactersitic	
MGMT+	68 (47)
MGMT-	76 (53)
IDH-1mut	19 (13)
IDH-1wt	125 (87)
MRI Charactersitic	
Median tumor volume (T1+contrast)	29.75 (IQR: 36.6)
Median edema volume (T2)	114 (IQR: 120.8)
Edema/Tumor Ratio <3	72 (50)
Edema/Tumor Ratio >3	72 (50)
Immunologica I Charactersitic (n=26)	
Lymphocytic infiltration (median/mm2)	0.18 (IQR: 0.15)
Granulocytic infiltration (median/mm2)	0.08 (IQR: 0.66)

### Association of Edema to Tumor Ratio With Patient Characteristics

According to the median preoperative MRI edema to tumor volume, patients were stratified into the high or low edema to tumor ratio groups. Patients of the high edema to tumor ratio group had 57% (n=41) female patients compared to 54% (n= 39) in the low edema to tumor cohort. No significant differences regarding sex were observed between both groups (p=0.86; 95%CI: 0.57-2.16; OR 1.1). In the high edema to tumor cohort, 35 (49%) patients were under the age of 60 compared to 29 (40%) patients in the low edema to tumor cohort. No significant differences regarding age were observed between both groups. Moreover, 31 (43%) patients with a high edema to tumor ratio and 32 patients (44%) with a low edema to tumor ratio presented with a median preoperative Karnofsky performance scale (KPS) of less than 80. The preoperative KPS was not associated with edema to tumor ratio (p=0.99; 95%CI:0.48-1.82; OR: 0.9); see **Table 2**.

**Table 2:** Uni- and multivariate analysis of juxtaposed characteristics according to edema to tumor ratio.

Variable(%)	Edema/Tumor ratio		Univariate			Multivariate		
	high *(n = 72)*	low *(n=72)*	*P* value	95% CI	OR	*P* value	95% CI	OR
Gender								
Male	41(57)	39 (54)	0.86	0.57-2.16	1.1	0.32	0.96-1.03	0.9
Female	31(43)	33 (46)	0.86	0.46-1.71	0.8			
Age (years)								
<60	35 (49)	29 (40)	0.40	0.72-2.71	1.4	1.01	0.98-1.01	0.9
≥60	37 (51)	43 (60)	0.40	0.36-1.37	0.7			
Karnofsky performance scale								
<80	31(43)	32 (44)	0.99	0.48-1.82	0.9	0.6	0.98-1.21	1.0
≥80	41(57)	40 (56)	0.99	0.54-2.04	1.0			
Dexamethasone preoperative								
yes	26 (36)	31 (43)	0.49	0.38-1.47	0.7	0.95	0.55-15.69	0.9
no	46 (64)	41 (57)	0.49	0.68-2.61	1.3			
Surgical Charactersitic								
Gross totaI resection	45 (62)	44 (61)	0.99	0.54-2.07	1.0	0.87	0.45-1.26	0.4
Partial resection	27 (38)	28 (39)	0.99	0.48-1.84	0.9			
Molecular Charactersitic								
MGMT+	38 (53)	30 (42)	0.40	0.36-1.37	0.7	0.85	0.57-1.92	1.0
MGMT-	34 (47)	42 (58)	0.40	0.72-2.72	1.4			
IDH-1mut	4 (6)	15 (21)	0.01	0.07-0.71	0.2	0.03	0.13-0.90	0.34
IDH-1wt	68 (94)	57 (79)	0.01	1.40-14.23	1.4			
Immunological Charactersitic (n=26)	*n=9*	*n=7*						
Median lymphocytic infiltration per mm2	0.15 (IQR: 0.18)	0.21(IQR: 0.13)	0.78	0.38-0.50	0.06			
Median granulocytic infiltration per mm2	0.06 (IQR: 0.16)	0.08 (IQR: 0.05)	0.74	0.10-0.14	0.02			

### Preoperative Dexamethasone Administration and Edema to Tumor Ratio

Patient medical charts showed preoperative dexamethasone (DEX) administration present in 26 patients (36%) in the high edema to tumor ratio cohort vs. 31 patients (43%) in the low edema to tumor ratio cohort. Preoperative presence of DEX was not associated with a high edema to tumor ratio (p=0.49; 95%CI: 0.38-1.47; OR: 0.7; **Table 2**).

### Association of Edema to Tumor Ratio With Operative Results

Gross total resection (GTR) was achieved in 45 patients (62%) in the high edema to tumor cohort and 44 patients (61%) in the low edema to tumor cohort. The operative result was independent of preoperative edema to tumor ratio (p=0.99; 95% 0.54-2.07; OR: 1.0; **Table 2**).

### Association of Edema to Tumor Ratio With Molecular Characteristics

In patients with a high edema to tumor ratio, the MGMT promotor was methylated (+) in 38 patients (53%) and in 30 patients (42%) of the low edema to tumor ratio cohort (p=0.40; 95%CI: 0.36-1.37; OR: 07). The MGMT promotor status was not significantly correlated with edema to tumor ratio. Isocitrate dehydrogenase 1 wildtype (IDH-1 wt) was observed in 68 patients (94%) with a high edema to tumor ratio and in 57 patients (79%) with a low edema to tumor ratio (p=0.01; 95%CI: 0.07-0.71, OR: 0.2 in univariate and p=0.03; 95%CI: 0.13-0.90, OR: 4.6 in multivariate analysis. (**Table 2**).

### Survival Analysis Depending on Edema to Tumor Volume

**Figure 3:**
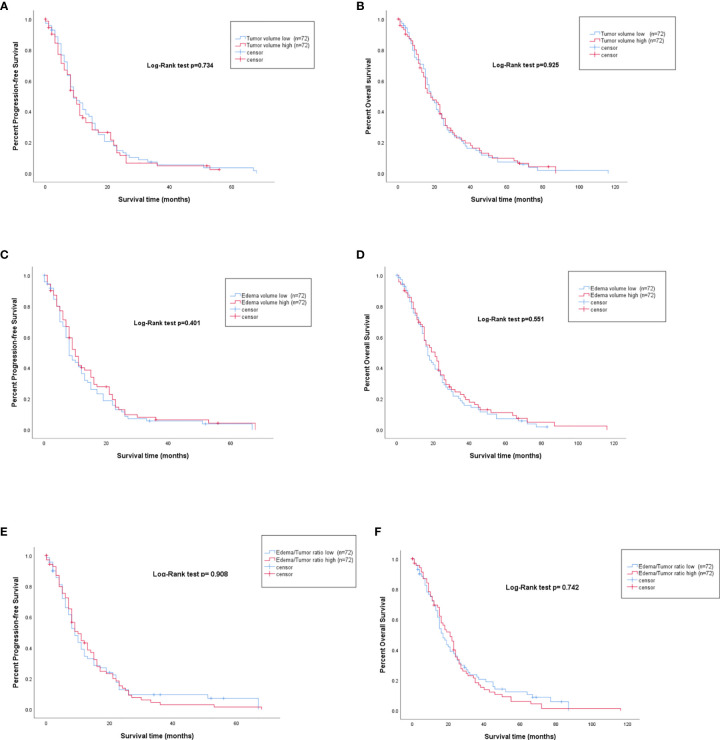
PFS and OS represented by Kaplan-Meier curves for high vs. low preoperative tumor volume **(A, B)**, high vs. low preoperative edema volume **(C, D)**, and high vs. low edema to tumor ratio **(E, F)**.

Volumetric analysis displayed a median tumor size of 29.75cm^3^ (IQR: 37.52). Patients with a tumor smaller than 29.75cm^3^ were defined as low tumor volume and patients with tumor size larger than 29.75cm^3^ as high tumor volume. Patients with low tumor volume had a median OS of 17 months (IQR: 19) and a PFS of 9 months (IQR: 12) compared to 16 months OS (IQR: 19.25) and 8 months PFS (IQR:11.75) in patients with large tumors, respectively. Preoperative volumetric tumor size was not associated with PFS (p=0.734) or OS (0.925; **Figure 3A, B**).

For the volumetric analysis of peritumoral edema, a median of 116.3cm^3^ (IQR: 120.8) was detected. Patients with peritumoral edema smaller than 116.3cm^3^ were defined as low edema volume and had a median OS of 16 months (IQR: 19.75) and a PFS of 8 months (IQR: 10.75) whereas patients with high edema volume had a median OS of 19 months (IQR: 19) and a median PFS of 9.5 months (IQR: 11). The volume of peritumoral edema was not associated with either PFS (p=0.55) or OS (p=0.40; **Figure 3C, D**).

To analyze the edema to tumor ratio, edema/tumor was calculated and a median of 3 was observed. Thus, patients with ratios under 3 were counted as having a low edema to tumor ratio and above 3 as having a high edema to tumor ratio. Patients with low edema to tumor ratio had a median PFS of 8 months (IQR: 12), compared to the PFS of 9 months (IQR: 11) in patients with high preoperative edema to tumor ratio (p=0.401). In terms of OS, patients with a low preoperative edema to tumor ratio displayed a median survival of 15 months (IQR: 21) compared with 19 months (IQR: 18) in patients with a high edema to tumor ratio (p=0.551; **Figures 3E, F**).

### Leukocytic Tumor Infiltration in Dependence of Edema to Tumor Ratio

In our cohort of 144 patients, immunohistochemistry was available in a total of 26 patients. In the high edema to tumor ratio group (n=9), median lymphocytic infiltration was 0.15 cells/mm^2^ (IQR: 0.18) vs. 0.21 cells/mm^2^ (IQR: 0.13) in the low edema to tumor ratio group (n=17) indicating that the lymphocytic tumor infiltration was not significantly correlated with edema to tumor ratio (p=0.78; 95%CI: 0.38-0.50; OR: 0.06). Furthermore, median myelocytic tumor infiltration was 0.06 cells/mm^2^ (IQR: 0.16) in the high edema to tumor ratio group vs. 0.08 cells/mm^2^ (IQR: 0.05) in the low edema to tumor ratio group. The myelocytic tumor infiltration was therefore not associated with edema to tumor ratio (p=0.74; 95% CI: 0.10-0.14; OR 0.02) (**Table 2**).

## Discussion

The major finding of our study is the significant correlation between IDH-1 status and the edema to tumor ratio. Patients harboring an IDH-1 wildtype glioblastoma presented with an increased preoperative edema to tumor ratio; however, further translational investigations are warranted to elucidate the underlaying mechanism. Furthermore, our immunohistochemistry analysis and its correlation with the edema to tumor ratio revealed that edema to tumor ratio had no significant association with lymphocytic and/or myelocytic tumor infiltration.

The interplay between cancer and immune cells is a major determinant in cancer progression and the immune system is emerging as a powerful prognostic marker and therapeutic target in neuro-oncology (10). Additionally, peritumoral edema frequently leads to neurological impairment and remains a challenging factor throughout treatment (3). Our study therefore analyzed a potential interaction between these factors.

Regarding the preoperative dexamethasone (DEX) treatment our study found no significant correlation between the extent of edema and DEX administration. Although dosage recommendations are lacking, the routinely used synthetic corticoid DEX is clinically established for edema treatment (11, 12). However, recent studies identified several mechanisms indicating that administration of DEX in patients with GBM may be not be beneficial; in fact, it may worsen the prognosis, decrease radio-sensitivity, and decreased immune infiltration is certain subtypes of GBM (3, 4, 13). Considering the missing association between the extent of tumor-edema and DEX dosage in our study, this finding supports the critical review of DEX administration in GBM patients, even more so in lesions that are not strategically located (motor or sensory cortex, language cortex, insula or basal ganglia), since several recently discovered mechanisms explain the unbeneficial impact of DEX on the outcome in GBM (3, 13).

Our study did not find a significant association between peritumoral edema or edema to tumor ratio with the extent of resection. Wu et al. recently described the negative impact of the extent of peritumoral edema (≥1 cm from tumor margin on axial MRI) on survival (6). Extensive peritumoral edema can lead to intraoperative challenges as it may obscure anatomical landmarks and complicate resection, which in turn contributes to poor survival (14, 15). Our finding does not support this proposal, since resection status was not associated with edema or edema to tumor ratio in our cohort. An important bias which could obscure our results is the inhomogeneous surgical management (5-ALA, intraoperative MRI etc.) paired with differences in surgeon experience. However, since we included the postoperative results in terms of gross vs subtotal resection we ensured to exclude a significant impact of this potential confounder.

One of the major findings of our analysis is the positive correlation between IDH-1 wildtype and a higher edema to tumor ratio. A few studies have investigated the association of IDH-1 status with morphological MRI analysis in low grade gliomas (16). IDH-1 mutated tumors present frequently with a unilateral pattern of growth, sharply defined tumor margins, homogeneous signal intensity, and less contrast enhancement on MRI. All these factors might contribute to the improved prognosis of patients with this subtype of GBM; however, an association between edema and IDH-1 is not described in the literature (16). The IDH-1 mutation leads to neo-enzymatic activity of the IDH-1 enzyme which drives the conversion of isocitrate into 2-hydroxyglutarate (2-HG), leading to a genome-wide histone- and DNA methylation alternations (17, 18). One of these consequences is the increased hypoxia-inducible factor 1-alpha (HIF-1α) that is frequently detected in patients with an IDH-1 mutation (19, 20). On the contrary, the proliferation rate (Ki-67) and vascular endothelial growth factor (VEGF) levels are significantly lower in IDH-1 mutated tumors (21, 22). However, little is known about the affinity for vasogenic edema in IDH-1 wildtype GBM (23). Our findings encourage further translational investigations into the mechanism of peritumoral edema in GBM wildtype.

Several studies have investigated the extent of peritumoral edema and correlated it with patient’s outcome but the results were conflicting and a consistent conclusion is therefore absent (1, 6, 24). In a multicenter analysis, Schoeneggers et al. identified edema as an independent prognostic factor for poor outcome in GBM (1). On the contrary, Lacroix et al. published their results including more than 400 GBM patients where the extent of peritumoral edema was not found to be an independent prognostic factor (25). In our study, neither tumor size, nor edema or a high edema to tumor ratio were associated with outcome. A possible explanation is the high-level of heterogeneity within the peritumoral edema tissue. Since the majority (>90%) of the tumors relapse in the peritumoral zone, the microenvironment with alternated tissue hemostasis may play a crucial role in recurrence, and the currently standardized quantitative measurements (T1+, T2, FLAIR etc.) do no capture molecular alternations in the peritumoral edema (26, 27). Radiomic approaches may complement current MRI imaging in future studies and elucidate pathological processes in peritumoral edema tissue.

Within this study, we addressed the effects of edema on immune infiltration (2). The assessment of immune infiltration is of paramount importance since the introduction of T cells with the expression of chimeric antigen receptors (CARs) directed against specific antigens (EGFRvIII-, HER2- and IL-13 Rα2 CAR T-cells) have initiated the era of personalized immunotherapy in GBM; and the results of several ongoing studies are eagerly anticipated (10, 28, 29). Berghoff et al. published their findings on CNS metastasis, where a high density of tumor-infiltrating lymphocytes (TIL) was more frequently observed in patients with low peritumoral edema, as compared to patients with high peritumoral edema; however, studies analysing this issue in GBMs are absent (30). In our analysis neither myelocytic nor lymphocytic infiltration was correlated with peritumoral edema. A possible explanation could be that the peritumoral microenvironment in brain tumor patients contains an array of other non-neoplastic cells, including vascular and other glial cells, all of which could contribute to edema formation (27). Although immunotherapy in general and CAR T administration in specific carries a risk of cerebral edema as a dreadful complication, our data implies that patients with a high edema to tumor ratio should not be excluded from further immunological studies (31, 32).

We did analyze a large number of patients with extensive radiological and immunohistological parameters, whereas histopathological analysis was only available in nearly 20% of the cases. As a limitation, the investigated cohort is from 2008-2013. Although all patients in our cohort received the temozolomide based radio-chemotherapy which still is the standard of care, novel therapies such as TTFields could not have been investigated. Our cohort is representative of GBM patients since the molecular profile is congruent with the literature (∼10% IDH mutated GBM in the literature and in 13% in our cohort) (18). As further limitation and potentially introducing a bias, this investigation was a single centre study and of retrospective design.

In conclusion, the present study found no association between the extent of edema and immunogenic infiltration; however, IDH wildtype GBM was found to be more likely associated with extensive peritumoral edema than IDH mutant GBM. Further translational investigations are necessary to evaluate the underlying mechanism and the clinical relevance of this observation.

## Data Availability Statement

The raw data supporting the conclusions of this article will be made available by the authors, without undue reservation.

## Ethics Statement

For this retrospective analysis, an ethical approval was obtained from the ethics committee of the University Hospital Frankfurt, Germany, (Identification number: 20-676). As a non-interventional single-center study, no patient consent was necessary.

## Author Contributions

DD collected the data and wrote the first draft. FG supervised the manuscript. All authors supplied additional information, edited the manuscript and contributed to critical review and revision of the manuscript. All authors contributed to the article and approved the submitted version.

## Conflict of Interest

The authors declare that the research was conducted in the absence of any commercial or financial relationships that could be construed as a potential conflict of interest.
